# Crystal structure of 15-(2-chloro­phen­yl)-6b-hy­droxy-17-methyl-6b,7,16,17-tetra­hydro-7,14a-methanona­phtho[1′,8′:1,2,3]pyrrolo­[3′,2′:8,8*a*]azuleno[5,6-*b*]quinolin-14(15*H*)-one

**DOI:** 10.1107/S2056989015024767

**Published:** 2015-12-31

**Authors:** J. M. Joseph, Vijayan Viswanathan, Devadasan Velmurugan

**Affiliations:** a Centre of Advanced Study in Crystallography and Biophysics, University of Madras, Guindy Campus, Chennai 600 025, India

**Keywords:** crystal structure, pyrrolidine derivative, hydrogen bonding

## Abstract

In the title compound, C_34_H_25_ClN_2_O_2_, the fused pyrrolidine ring adopts an envelope conformation with the N atom as the flap. The two adjacent cyclo­pentane rings also adopt envelope conformations. The mean plane of the pyrrolidine ring makes dihedral angles of 40.53 (10) and 80.23 (10)° with the mean planes of the cyclo­pentane rings. The dihedral angle between the mean planes of the cyclo­pentane rings is 46.71 (9)°. An intra­molecular O—H⋯N hydrogen bond is observed. In the crystal, mol­ecules are linked by C—H⋯O, C—H⋯N and C—H⋯π inter­actions, forming a layer parallel to (10-2).

## Related literature   

For biological activities of pyrrolidine derivatives, see: Aravindan *et al.* (2004 ▸); Gayathri *et al.* (2005 ▸); Seki *et al.* (2013 ▸); Li & Xu (2004 ▸); Arun *et al.* (2014 ▸); Govind *et al.* (2003 ▸); Nirmala *et al.* (2009 ▸); Sharma & Soman (2015 ▸); Bellina & Rossi (2006 ▸); Babu *et al.* (2012 ▸). For related structures, see: Savithri *et al.* (2014 ▸).
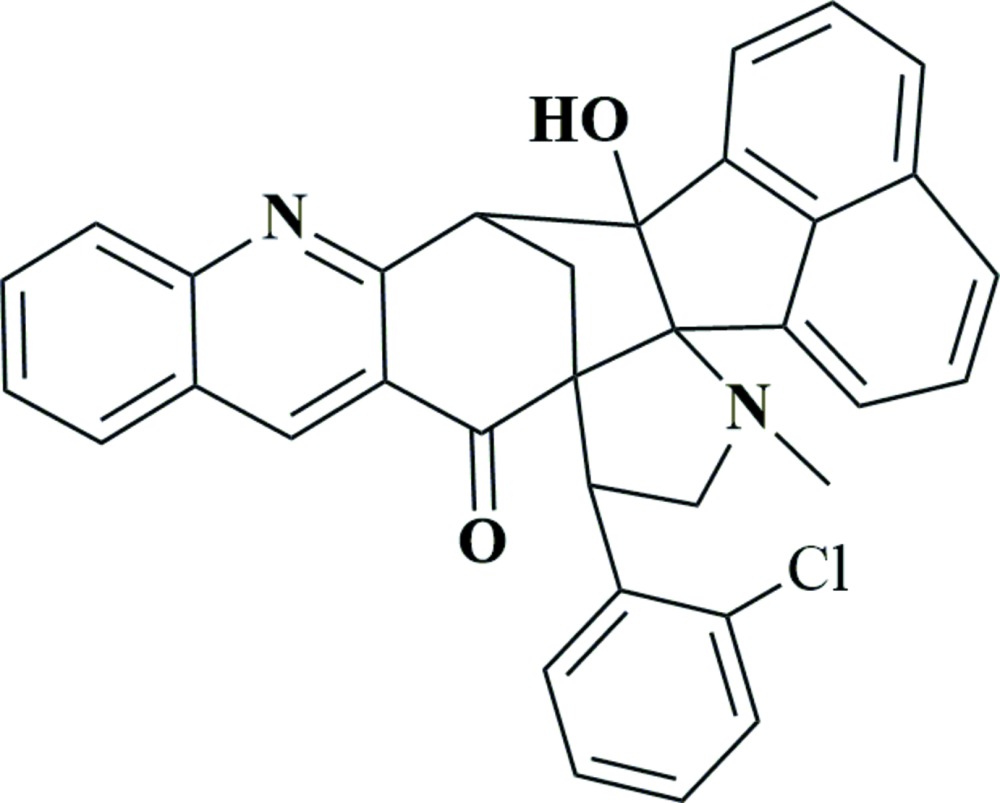


## Experimental   

### Crystal data  

C_34_H_25_ClN_2_O_2_
*M* *_r_* = 529.01
Monoclinic, 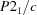

*a* = 11.1328 (2) Å
*b* = 13.0756 (3) Å
*c* = 19.0866 (4) Å
β = 103.738 (1)°
*V* = 2698.91 (10) Å^3^
*Z* = 4
Mo *K*α radiation
μ = 0.18 mm^−1^
*T* = 293 K
0.30 × 0.25 × 0.20 mm

### Data collection  

Bruker SMART APEXII area-detector diffractometer
Absorption correction: multi-scan (*SADABS*; Bruker, 2008 ▸) *T*_min_ = 0.949, *T*_max_ = 0.966
25834 measured reflections
6713 independent reflections
4796 reflections with *I* > 2σ(*I*)
*R*_int_ = 0.022

### Refinement  

*R*[*F* ^2^ > 2σ(*F* ^2^)] = 0.049
*wR*(*F* ^2^) = 0.139
*S* = 1.05
6713 reflections
354 parameters
H-atom parameters constrained
Δρ_max_ = 0.40 e Å^−3^
Δρ_min_ = −0.58 e Å^−3^

### 

Data collection: *APEX2* (Bruker, 2008 ▸); cell refinement: *SAINT* (Bruker, 2008 ▸); data reduction: *SAINT*; program(s) used to solve structure: *SHELXS97* (Sheldrick, 2008 ▸); program(s) used to refine structure: *SHELXL2014* (Sheldrick, 2015 ▸); molecular graphics: *ORTEP-3 for Windows* (Farrugia, 2012 ▸); software used to prepare material for publication: *PLATON* (Spek, 2009 ▸).

## Supplementary Material

Crystal structure: contains datablock(s) global, I. DOI: 10.1107/S2056989015024767/is5437sup1.cif

Structure factors: contains datablock(s) I. DOI: 10.1107/S2056989015024767/is5437Isup2.hkl

**Fig. 1.**
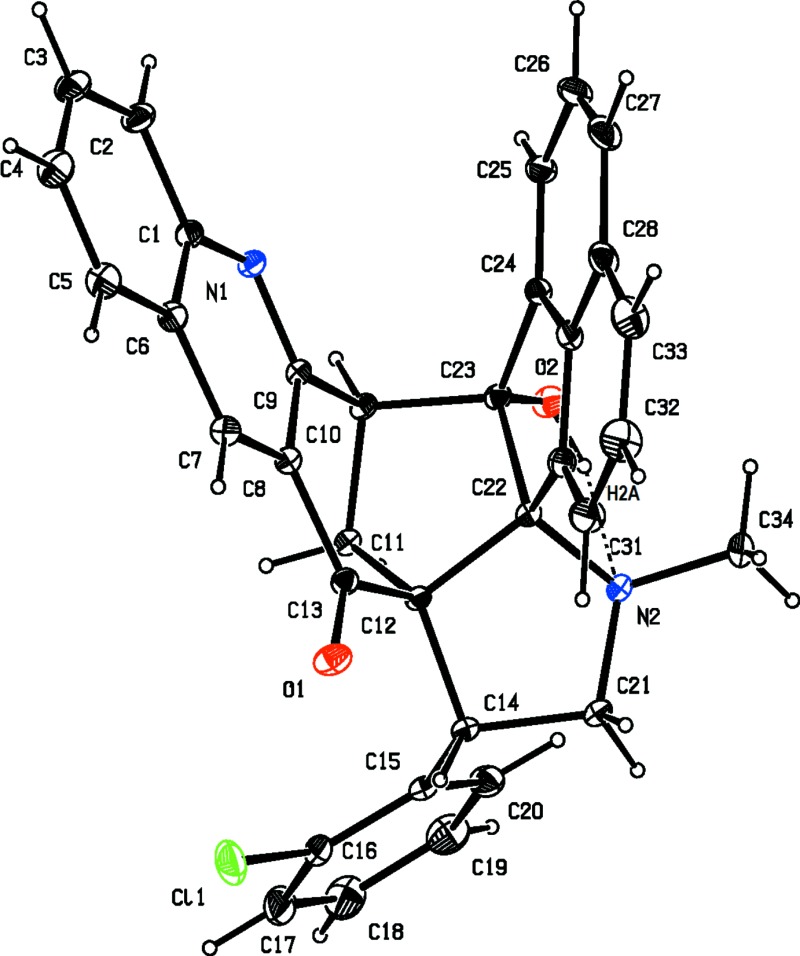
The molecular structure of the title compound, showing the atomic numbering and displacement ellipsoids drawn at 20% probability level.

. DOI: 10.1107/S2056989015024767/is5437fig1.tif
The mol­ecular structure of the title compound, showing the atomic numbering and displacement ellipsoids drawn at 20% probability level.

**Fig. 2.**
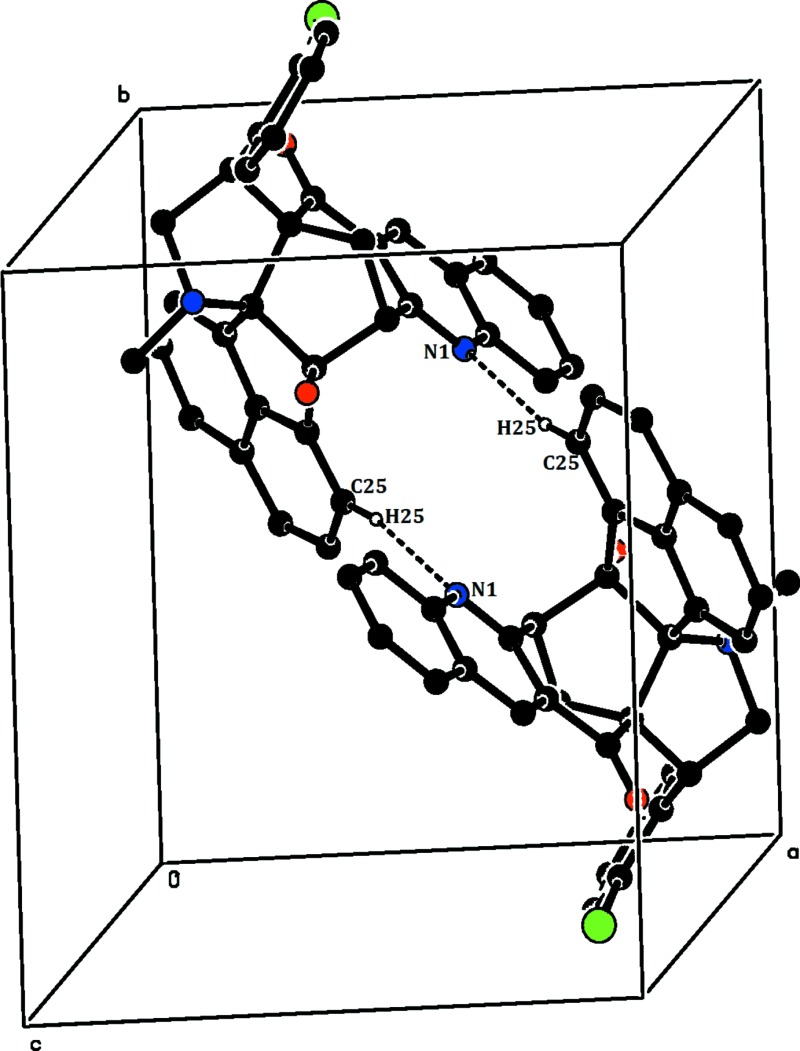
A packing diagram of the title compound viewed approximately down the c axis, showing the R22(14) ring motif formed by C—H···N hydrogen bonds (dashed lines). H-atoms not involved in hydrogen bonds have been excluded for clarity.

**Fig. 3.**
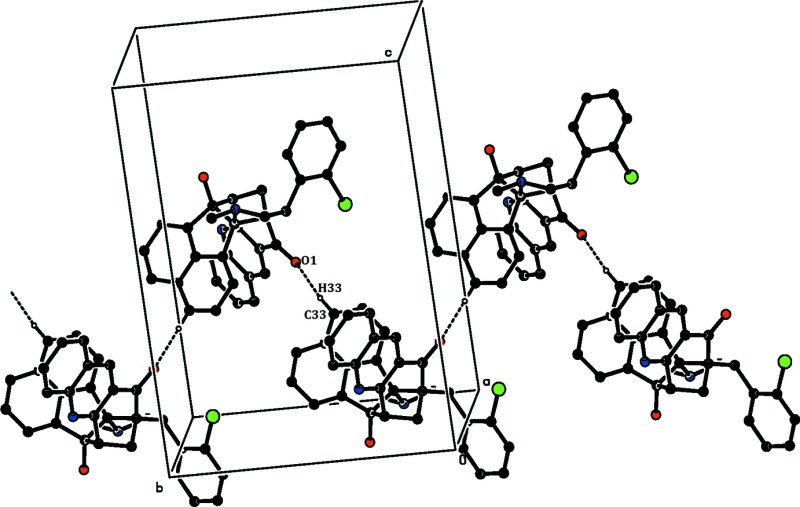
A packing diagram of the title compound viewed approximately down the b axis. C—H···O hydrogen bonds are indicated by dashed lines. H atoms not involved in the hydrogen bonds have been excluded for clarity.

Click here for additional data file.c . DOI: 10.1107/S2056989015024767/is5437fig2.tif
A packing diagram of the title compound viewed approximately down the *c* axis, showing the 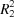
(14) ring motif formed by C—H⋯N hydrogen bonds (dashed lines). H-atoms not involved in hydrogen bonds have been excluded for clarity.

b . DOI: 10.1107/S2056989015024767/is5437fig3.tif
A packing diagram of the title compound viewed approximately down the *b* axis. C—H⋯O hydrogen bonds are indicated by dashed lines. H atoms not involved in the hydrogen bonds have been excluded for clarity.

**Fig. 4.**
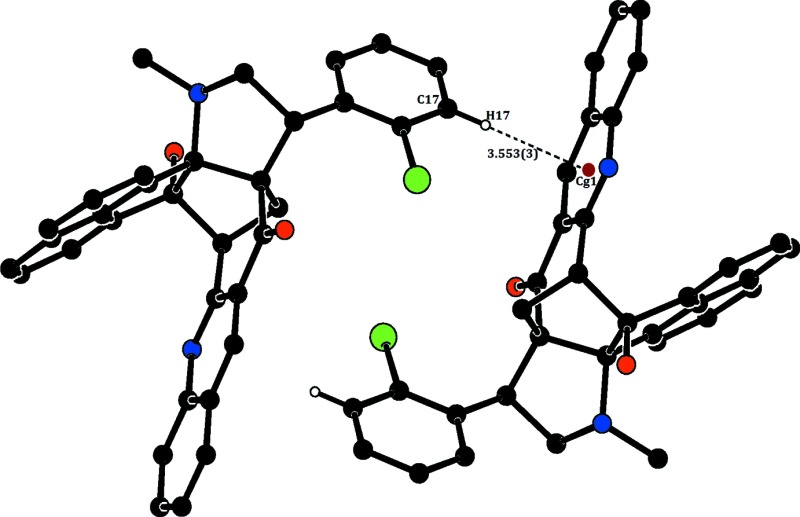
A packing diagram of the title compound showing a C—H···π interaction (dashed line).

. DOI: 10.1107/S2056989015024767/is5437fig4.tif
A packing diagram of the title compound showing a C—H⋯π inter­action (dashed line).

CCDC reference: 1444162

Additional supporting information:  crystallographic information; 3D view; checkCIF report

## Figures and Tables

**Table 1:** Hydrogen-bond geometry (Å, °) *Cg*1 is the centroid of the N1/C1/C6–C9 ring.

*D*—H⋯*A*	*D*—H	H⋯*A*	*D*⋯*A*	*D*—H⋯*A*
O2—H2*A*⋯N2	0.82	2.12	2.664 (2)	124
C25—H25⋯N1^i^	0.93	2.59	3.503 (2)	169
C33—H33⋯O1^ii^	0.93	2.40	3.212 (3)	146
C17—H17⋯*Cg*1^iii^	0.93	2.73	3.553 (3)	147

Symmetry codes: (i) 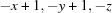; (ii) 
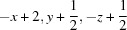
; (iii) 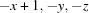.

## supplementary crystallographic information

### S1. Comment

Pyrrolidine compounds are often encountered in pharmacologically relevant alkaloids (Aravindan *et al.*, 2004). Optically active pyrrolidines have been used as intermediates, chiral ligands or auxiliaries in controlled asymmetric synthesis (Savithri *et al.*, 2014). Synthetic spiro pyrrolidine derivatives exhibit activity against the aldose reductase enzyme, which controls influenza virus (Gayathri *et al.*, 2005) and also pyrrolidine compounds are reported to exhibit ischemic stroke (Seki *et al.*, 2013), anti-inflammatory (Li & Xu, 2004), antitumor (Arun *et al.*, 2014), antimicrobial, antifungal (Govind *et al.*, 2003), antibiotic (Nirmala *et al.*, 2009) and anti-diabetic (Sharma & Soman, 2015) activities and inhibition of retroviral reverse transcriptases [i.e., human immunodeficiency virus type 1 (HIV-1)], cellular DNA polymerases and protein kinases (Bellina & Rossi, 2006). They are also anticonvulsants, sphingosine-1-phosphate (S1P) receptor agonists, malic enzyme inhibitors, ketoamide-based cathepsin K inhibitors and human melanocortin-4 receptor agonists (Babu *et al.*, 2012). In view of the above biological importance, the crystal structure of the title compound was determined.

In the title molecule, one cyclopentane ring I (C10–C12/C22/C23) is fused with the other cyclopentane ring II (C22–C24/C29/C30) of the acenaphthylene ring system (C22–C33). The pyrrolidine ring (C12/C14/C21/N2/C22) is fused with the cyclopentane ring I, and adopts an envelope conformation with atom N2 as the flap atom deviating by 0.5765 (2) Å from the mean plane defined by the other atoms (C12/C14/C21/C22). The two cyclopentane rings I and II adopt envelope conformations with atoms C11 and C22 as the flap atoms, respectively, deviating by 0.7033 and 0.1765 Å from the mean plane. The mean plane of the pyrrolidine ring makes dihedral angles of 40.53 (10) and 80.23 (10)° with the mean planes of the cyclopentane rings I and II, respectively. The mean plane of the pyrrolidine ring makes dihedral angles of 82.04 (8) and 68.25 (9)° with the mean plane of the acenapthylene and phenyl (C8–C13) ring systems, respectively. The mean plane of the cyclopentane ring I makes a dihedral angle of 46.71 (9)° with the mean plane of the cyclopentane ring II. The mean plane of the cyclopentane ring makes dihedral angles of 47.85 (7) and 87.06 (9)° with the mean plane of the acenaphthylene ring system and the phenyl ring, respectively.

In the crystal, a pair of C—H···N interactions (Table 1) show an *R*_2_^2^(14) ring (Fig. 2). In addition, a C—H···O hydrogen bond links the symmetry-related molecules, forming a helical chain running along the *b* axis (Fig. 3). The two molecules are also held together by a C—H···π interaction (Fig. 4).

### S2. Experimental

A mixture of (*E*)-2-(2-chlorobenzylidene)-3,4-dihydroacridin-1(2*H*)-one (1 mmol), acenaphthoquinone (1 mmol) and sarcosine (1.5 mmol) was heated to reflux in toluene (3 ml) for 10 h. After completion of the reaction as evident from TLC, the reaction mixture was extracted with ethyl acetate (2 × 20 ml), washed with water (2 × 10 ml), dried over anhydrous Na_2_SO_4_ and concentrated under reduced pressure, subjected to column chromatography using petroleum ether-AcOEt (5:1 *v*/*v*) as eluent to obtain pure product. Single crystals suitable for X-ray diffraction were prepared by slow evaporation of a solution of the title compound in ethanol at room temperature.

### S3. Refinement

The hydrogen atoms were placed in calculated positions with C—H = 0.93–0.98 Å and O—H = 0.82 Å, and were refined in a riding model with *U*_iso_(H) = 1.5*U*_eq_(C_methyl_, O) or 1.2*U*_eq_(C).

### Crystal data

**Table:**

C_34_H_25_ClN_2_O_2_	*F*(000) = 1104
*M**_r_* = 529.01	*D*_x_ = 1.302 Mg m^−^^3^
Monoclinic, *P*2_1_/*c*	Mo *K*α radiation, λ = 0.71073 Å
*a* = 11.1328 (2) Å	Cell parameters from 6713 reflections
*b* = 13.0756 (3) Å	θ = 1.9–28.3°
*c* = 19.0866 (4) Å	µ = 0.18 mm^−^^1^
β = 103.738 (1)°	*T* = 293 K
*V* = 2698.91 (10) Å^3^	Block, colourless
*Z* = 4	0.30 × 0.25 × 0.20 mm

### Data collection

**Table:**

Bruker SMART APEXII area-detector diffractometer	4796 reflections with *I* > 2σ(*I*)
ω and φ scans	*R*_int_ = 0.022
Absorption correction: multi-scan (*SADABS*; Bruker, 2008)	θ_max_ = 28.3°, θ_min_ = 1.9°
*T*_min_ = 0.949, *T*_max_ = 0.966	*h* = −14→13
25834 measured reflections	*k* = −12→17
6713 independent reflections	*l* = −23→25

### Refinement

**Table:**

Refinement on *F*^2^	0 restraints
Least-squares matrix: full	Hydrogen site location: inferred from neighbouring sites
*R*[*F*^2^ > 2σ(*F*^2^)] = 0.049	H-atom parameters constrained
*wR*(*F*^2^) = 0.139	*w* = 1/[σ^2^(*F*_o_^2^) + (0.0597*P*)^2^ + 0.848*P*] where *P* = (*F*_o_^2^ + 2*F*_c_^2^)/3
*S* = 1.05	(Δ/σ)_max_ = 0.001
6713 reflections	Δρ_max_ = 0.40 e Å^−^^3^
354 parameters	Δρ_min_ = −0.58 e Å^−^^3^

### Special details

**Table:**

Geometry. All esds (except the esd in the dihedral angle between two l.s. planes) are estimated using the full covariance matrix. The cell esds are taken into account individually in the estimation of esds in distances, angles and torsion angles; correlations between esds in cell parameters are only used when they are defined by crystal symmetry. An approximate (isotropic) treatment of cell esds is used for estimating esds involving l.s. planes.

### Fractional atomic coordinates and isotropic or equivalent isotropic displacement parameters (Å^2^)

**Table:**

	*x*	*y*	*z*	*U*_iso_*/*U*_eq_	
C1	0.48949 (15)	0.35516 (13)	0.16996 (8)	0.0410 (4)	
C2	0.40120 (18)	0.42324 (15)	0.18580 (11)	0.0561 (5)	
H2	0.3615	0.4701	0.1514	0.067*	
C3	0.3743 (2)	0.41996 (17)	0.25204 (12)	0.0673 (6)	
H3	0.3162	0.4650	0.2624	0.081*	
C4	0.4328 (2)	0.34998 (18)	0.30440 (11)	0.0690 (6)	
H4	0.4126	0.3488	0.3490	0.083*	
C5	0.5182 (2)	0.28387 (16)	0.29125 (10)	0.0587 (5)	
H5	0.5564	0.2377	0.3266	0.070*	
C6	0.54938 (16)	0.28509 (13)	0.22343 (8)	0.0433 (4)	
C7	0.63722 (15)	0.21934 (13)	0.20603 (8)	0.0423 (4)	
H7	0.6800	0.1734	0.2402	0.051*	
C8	0.65996 (13)	0.22281 (12)	0.13869 (8)	0.0352 (3)	
C9	0.59359 (13)	0.29450 (11)	0.08764 (7)	0.0327 (3)	
C10	0.61453 (13)	0.29544 (12)	0.01248 (7)	0.0338 (3)	
H10	0.5419	0.3218	−0.0224	0.041*	
C11	0.64578 (13)	0.18722 (12)	−0.00706 (8)	0.0355 (3)	
H11A	0.6496	0.1826	−0.0572	0.043*	
H11B	0.5861	0.1381	0.0021	0.043*	
C12	0.77426 (13)	0.17073 (11)	0.04414 (8)	0.0337 (3)	
C13	0.75273 (14)	0.15339 (12)	0.11889 (8)	0.0379 (3)	
C14	0.86006 (14)	0.09018 (12)	0.02079 (9)	0.0407 (4)	
H14	0.8876	0.0415	0.0604	0.049*	
C15	0.79749 (15)	0.03145 (13)	−0.04622 (9)	0.0449 (4)	
C16	0.72850 (17)	−0.05621 (15)	−0.04343 (11)	0.0560 (5)	
C17	0.6726 (2)	−0.1110 (2)	−0.10403 (15)	0.0814 (7)	
H17	0.6286	−0.1702	−0.0997	0.098*	
C18	0.6820 (3)	−0.0784 (2)	−0.17021 (16)	0.0933 (9)	
H18	0.6450	−0.1156	−0.2112	0.112*	
C19	0.7460 (3)	0.0090 (2)	−0.17633 (13)	0.0830 (7)	
H19	0.7506	0.0325	−0.2216	0.100*	
C20	0.8039 (2)	0.06261 (16)	−0.11492 (11)	0.0613 (5)	
H20	0.8485	0.1212	−0.1198	0.074*	
C21	0.97124 (16)	0.15380 (13)	0.01258 (11)	0.0514 (4)	
H21A	1.0350	0.1541	0.0572	0.062*	
H21B	1.0059	0.1266	−0.0256	0.062*	
C22	0.83991 (13)	0.27695 (12)	0.04290 (8)	0.0368 (3)	
C23	0.73303 (14)	0.35448 (12)	0.00747 (8)	0.0356 (3)	
C24	0.75807 (15)	0.44880 (12)	0.05354 (9)	0.0401 (3)	
C25	0.70176 (18)	0.54227 (13)	0.04710 (10)	0.0505 (4)	
H25	0.6400	0.5590	0.0066	0.061*	
C26	0.7405 (2)	0.61256 (15)	0.10406 (12)	0.0630 (5)	
H26	0.7027	0.6764	0.1005	0.076*	
C27	0.8304 (2)	0.59131 (16)	0.16375 (13)	0.0667 (6)	
H27	0.8525	0.6403	0.1998	0.080*	
C28	0.89118 (18)	0.49524 (15)	0.17184 (11)	0.0558 (5)	
C29	0.85176 (15)	0.42601 (13)	0.11493 (9)	0.0431 (4)	
C30	0.90043 (15)	0.32693 (13)	0.11448 (9)	0.0447 (4)	
C31	0.99033 (18)	0.29500 (17)	0.17236 (11)	0.0635 (5)	
H31	1.0238	0.2297	0.1735	0.076*	
C32	1.0311 (2)	0.3634 (2)	0.23032 (13)	0.0821 (7)	
H32	1.0919	0.3419	0.2699	0.098*	
C33	0.9850 (2)	0.4597 (2)	0.23037 (13)	0.0767 (7)	
H33	1.0154	0.5026	0.2694	0.092*	
C34	1.0217 (2)	0.33226 (17)	−0.00314 (15)	0.0733 (6)	
H34A	0.9861	0.3991	−0.0134	0.110*	
H34B	1.0668	0.3148	−0.0386	0.110*	
H34C	1.0768	0.3321	0.0439	0.110*	
N1	0.51194 (12)	0.35848 (10)	0.10248 (7)	0.0385 (3)	
N2	0.92281 (13)	0.25697 (11)	−0.00534 (8)	0.0476 (3)	
O1	0.80595 (12)	0.08859 (10)	0.16014 (7)	0.0587 (4)	
O2	0.72880 (12)	0.37212 (10)	−0.06641 (6)	0.0493 (3)	
H2A	0.7820	0.3376	−0.0785	0.074*	
Cl1	0.71190 (7)	−0.10108 (5)	0.03928 (4)	0.0892 (2)	

### Atomic displacement parameters (Å^2^)

**Table:**

	*U*^11^	*U*^22^	*U*^33^	*U*^12^	*U*^13^	*U*^23^
C1	0.0437 (8)	0.0415 (9)	0.0410 (8)	0.0001 (7)	0.0162 (7)	−0.0055 (7)
C2	0.0609 (11)	0.0548 (11)	0.0601 (11)	0.0129 (9)	0.0294 (9)	−0.0031 (9)
C3	0.0781 (14)	0.0662 (13)	0.0708 (13)	0.0107 (11)	0.0438 (11)	−0.0124 (11)
C4	0.0904 (16)	0.0785 (15)	0.0503 (11)	0.0004 (12)	0.0410 (11)	−0.0097 (10)
C5	0.0772 (13)	0.0654 (13)	0.0383 (9)	−0.0001 (10)	0.0233 (9)	−0.0040 (8)
C6	0.0515 (9)	0.0458 (10)	0.0345 (8)	−0.0025 (7)	0.0142 (7)	−0.0052 (7)
C7	0.0483 (9)	0.0441 (9)	0.0333 (8)	0.0027 (7)	0.0076 (6)	0.0019 (6)
C8	0.0346 (7)	0.0376 (8)	0.0326 (7)	0.0017 (6)	0.0064 (5)	0.0002 (6)
C9	0.0305 (7)	0.0355 (8)	0.0322 (7)	0.0022 (6)	0.0076 (5)	0.0000 (6)
C10	0.0313 (7)	0.0399 (8)	0.0295 (7)	0.0077 (6)	0.0060 (5)	0.0026 (6)
C11	0.0327 (7)	0.0399 (8)	0.0343 (7)	0.0039 (6)	0.0086 (6)	−0.0018 (6)
C12	0.0299 (7)	0.0333 (8)	0.0383 (7)	0.0058 (6)	0.0088 (6)	−0.0002 (6)
C13	0.0348 (7)	0.0388 (8)	0.0386 (8)	0.0044 (6)	0.0058 (6)	0.0030 (6)
C14	0.0373 (8)	0.0351 (8)	0.0521 (9)	0.0091 (6)	0.0154 (7)	0.0010 (7)
C15	0.0416 (9)	0.0392 (9)	0.0589 (10)	0.0088 (7)	0.0216 (7)	−0.0060 (7)
C16	0.0469 (10)	0.0512 (11)	0.0772 (13)	−0.0024 (8)	0.0294 (9)	−0.0100 (9)
C17	0.0648 (14)	0.0767 (16)	0.109 (2)	−0.0227 (12)	0.0341 (13)	−0.0380 (14)
C18	0.0830 (18)	0.108 (2)	0.0918 (19)	−0.0133 (16)	0.0271 (15)	−0.0495 (17)
C19	0.0958 (18)	0.098 (2)	0.0607 (13)	0.0095 (16)	0.0300 (12)	−0.0200 (13)
C20	0.0762 (13)	0.0543 (12)	0.0616 (12)	0.0054 (10)	0.0324 (10)	−0.0078 (9)
C21	0.0378 (9)	0.0442 (10)	0.0767 (12)	0.0065 (7)	0.0228 (8)	−0.0055 (9)
C22	0.0321 (7)	0.0342 (8)	0.0452 (8)	0.0039 (6)	0.0116 (6)	0.0004 (6)
C23	0.0398 (8)	0.0360 (8)	0.0332 (7)	0.0057 (6)	0.0130 (6)	0.0030 (6)
C24	0.0458 (9)	0.0339 (8)	0.0448 (8)	0.0008 (6)	0.0194 (7)	0.0023 (6)
C25	0.0630 (11)	0.0374 (9)	0.0572 (10)	0.0063 (8)	0.0265 (9)	0.0053 (8)
C26	0.0865 (15)	0.0347 (10)	0.0769 (14)	0.0018 (9)	0.0372 (12)	−0.0027 (9)
C27	0.0848 (15)	0.0466 (12)	0.0748 (14)	−0.0192 (11)	0.0313 (12)	−0.0211 (10)
C28	0.0586 (11)	0.0490 (11)	0.0603 (11)	−0.0160 (9)	0.0153 (9)	−0.0131 (9)
C29	0.0433 (9)	0.0381 (9)	0.0494 (9)	−0.0067 (7)	0.0139 (7)	−0.0034 (7)
C30	0.0347 (8)	0.0431 (9)	0.0536 (10)	−0.0012 (7)	0.0049 (7)	−0.0025 (7)
C31	0.0455 (10)	0.0602 (12)	0.0728 (13)	0.0014 (9)	−0.0101 (9)	−0.0048 (10)
C32	0.0606 (13)	0.0888 (18)	0.0769 (15)	−0.0046 (12)	−0.0230 (11)	−0.0104 (13)
C33	0.0682 (14)	0.0793 (17)	0.0717 (14)	−0.0223 (12)	−0.0053 (11)	−0.0249 (12)
C34	0.0599 (13)	0.0560 (12)	0.1193 (19)	−0.0101 (10)	0.0518 (13)	−0.0053 (12)
N1	0.0384 (7)	0.0403 (7)	0.0391 (7)	0.0067 (5)	0.0138 (5)	0.0005 (5)
N2	0.0405 (7)	0.0404 (8)	0.0696 (9)	0.0018 (6)	0.0285 (7)	−0.0013 (7)
O1	0.0630 (8)	0.0633 (8)	0.0494 (7)	0.0281 (7)	0.0123 (6)	0.0195 (6)
O2	0.0607 (8)	0.0549 (8)	0.0384 (6)	0.0110 (6)	0.0235 (5)	0.0085 (5)
Cl1	0.1012 (5)	0.0769 (4)	0.1044 (5)	−0.0282 (3)	0.0540 (4)	0.0057 (3)

### Geometric parameters (Å, º)

**Table:**

C1—N1	1.3697 (19)	C17—H17	0.9300
C1—C2	1.411 (2)	C18—C19	1.366 (4)
C1—C6	1.415 (2)	C18—H18	0.9300
C2—C3	1.367 (3)	C19—C20	1.386 (3)
C2—H2	0.9300	C19—H19	0.9300
C3—C4	1.397 (3)	C20—H20	0.9300
C3—H3	0.9300	C21—N2	1.463 (2)
C4—C5	1.352 (3)	C21—H21A	0.9700
C4—H4	0.9300	C21—H21B	0.9700
C5—C6	1.418 (2)	C22—N2	1.4736 (19)
C5—H5	0.9300	C22—C30	1.520 (2)
C6—C7	1.400 (2)	C22—C23	1.587 (2)
C7—C8	1.368 (2)	C23—O2	1.4187 (17)
C7—H7	0.9300	C23—C24	1.502 (2)
C8—C9	1.426 (2)	C24—C25	1.366 (2)
C8—C13	1.490 (2)	C24—C29	1.403 (2)
C9—N1	1.3148 (18)	C25—C26	1.411 (3)
C9—C10	1.5071 (19)	C25—H25	0.9300
C10—C11	1.524 (2)	C26—C27	1.355 (3)
C10—C23	1.551 (2)	C26—H26	0.9300
C10—H10	0.9800	C27—C28	1.418 (3)
C11—C12	1.544 (2)	C27—H27	0.9300
C11—H11A	0.9700	C28—C29	1.402 (2)
C11—H11B	0.9700	C28—C33	1.414 (3)
C12—C13	1.519 (2)	C29—C30	1.405 (2)
C12—C14	1.556 (2)	C30—C31	1.368 (2)
C12—C22	1.572 (2)	C31—C32	1.411 (3)
C13—O1	1.2113 (19)	C31—H31	0.9300
C14—C15	1.512 (2)	C32—C33	1.359 (3)
C14—C21	1.530 (2)	C32—H32	0.9300
C14—H14	0.9800	C33—H33	0.9300
C15—C16	1.388 (3)	C34—N2	1.470 (2)
C15—C20	1.391 (3)	C34—H34A	0.9600
C16—C17	1.377 (3)	C34—H34B	0.9600
C16—Cl1	1.735 (2)	C34—H34C	0.9600
C17—C18	1.361 (4)	O2—H2A	0.8200
			
N1—C1—C2	118.26 (15)	C18—C19—C20	119.9 (2)
N1—C1—C6	122.61 (14)	C18—C19—H19	120.1
C2—C1—C6	119.12 (15)	C20—C19—H19	120.1
C3—C2—C1	119.67 (19)	C19—C20—C15	122.1 (2)
C3—C2—H2	120.2	C19—C20—H20	119.0
C1—C2—H2	120.2	C15—C20—H20	119.0
C2—C3—C4	120.98 (18)	N2—C21—C14	105.25 (13)
C2—C3—H3	119.5	N2—C21—H21A	110.7
C4—C3—H3	119.5	C14—C21—H21A	110.7
C5—C4—C3	121.00 (17)	N2—C21—H21B	110.7
C5—C4—H4	119.5	C14—C21—H21B	110.7
C3—C4—H4	119.5	H21A—C21—H21B	108.8
C4—C5—C6	119.86 (19)	N2—C22—C30	115.35 (13)
C4—C5—H5	120.1	N2—C22—C12	102.42 (12)
C6—C5—H5	120.1	C30—C22—C12	118.34 (13)
C7—C6—C1	117.54 (14)	N2—C22—C23	111.38 (12)
C7—C6—C5	123.10 (16)	C30—C22—C23	103.64 (12)
C1—C6—C5	119.36 (16)	C12—C22—C23	105.46 (11)
C8—C7—C6	119.86 (15)	O2—C23—C24	113.97 (13)
C8—C7—H7	120.1	O2—C23—C10	108.31 (12)
C6—C7—H7	120.1	C24—C23—C10	114.53 (12)
C7—C8—C9	118.84 (14)	O2—C23—C22	111.83 (12)
C7—C8—C13	120.81 (14)	C24—C23—C22	105.01 (12)
C9—C8—C13	120.35 (13)	C10—C23—C22	102.61 (11)
N1—C9—C8	122.93 (13)	C25—C24—C29	119.96 (16)
N1—C9—C10	118.10 (12)	C25—C24—C23	131.66 (16)
C8—C9—C10	118.93 (12)	C29—C24—C23	108.20 (13)
C9—C10—C11	108.73 (12)	C24—C25—C26	117.63 (18)
C9—C10—C23	112.88 (12)	C24—C25—H25	121.2
C11—C10—C23	101.55 (11)	C26—C25—H25	121.2
C9—C10—H10	111.1	C27—C26—C25	122.97 (19)
C11—C10—H10	111.1	C27—C26—H26	118.5
C23—C10—H10	111.1	C25—C26—H26	118.5
C10—C11—C12	101.87 (11)	C26—C27—C28	120.76 (18)
C10—C11—H11A	111.4	C26—C27—H27	119.6
C12—C11—H11A	111.4	C28—C27—H27	119.6
C10—C11—H11B	111.4	C29—C28—C33	115.85 (19)
C12—C11—H11B	111.4	C29—C28—C27	115.74 (18)
H11A—C11—H11B	109.3	C33—C28—C27	128.39 (19)
C13—C12—C11	106.63 (11)	C28—C29—C24	122.93 (16)
C13—C12—C14	114.36 (12)	C28—C29—C30	123.36 (17)
C11—C12—C14	116.91 (12)	C24—C29—C30	113.70 (15)
C13—C12—C22	108.88 (12)	C31—C30—C29	119.05 (17)
C11—C12—C22	103.37 (11)	C31—C30—C22	132.67 (17)
C14—C12—C22	105.93 (11)	C29—C30—C22	108.22 (14)
O1—C13—C8	121.52 (14)	C30—C31—C32	118.5 (2)
O1—C13—C12	123.58 (14)	C30—C31—H31	120.8
C8—C13—C12	114.89 (12)	C32—C31—H31	120.8
C15—C14—C21	114.25 (14)	C33—C32—C31	122.4 (2)
C15—C14—C12	113.27 (13)	C33—C32—H32	118.8
C21—C14—C12	103.17 (12)	C31—C32—H32	118.8
C15—C14—H14	108.6	C32—C33—C28	120.81 (19)
C21—C14—H14	108.6	C32—C33—H33	119.6
C12—C14—H14	108.6	C28—C33—H33	119.6
C16—C15—C20	115.53 (17)	N2—C34—H34A	109.5
C16—C15—C14	122.33 (16)	N2—C34—H34B	109.5
C20—C15—C14	122.14 (17)	H34A—C34—H34B	109.5
C17—C16—C15	122.8 (2)	N2—C34—H34C	109.5
C17—C16—Cl1	117.64 (17)	H34A—C34—H34C	109.5
C15—C16—Cl1	119.60 (15)	H34B—C34—H34C	109.5
C18—C17—C16	119.9 (2)	C9—N1—C1	118.18 (13)
C18—C17—H17	120.1	C21—N2—C34	112.26 (14)
C16—C17—H17	120.1	C21—N2—C22	105.56 (13)
C17—C18—C19	119.9 (2)	C34—N2—C22	116.07 (14)
C17—C18—H18	120.1	C23—O2—H2A	109.5
C19—C18—H18	120.1		
			
N1—C1—C2—C3	178.26 (18)	C13—C12—C22—C23	−98.81 (13)
C6—C1—C2—C3	−0.5 (3)	C11—C12—C22—C23	14.28 (14)
C1—C2—C3—C4	−0.1 (3)	C14—C12—C22—C23	137.76 (12)
C2—C3—C4—C5	0.4 (4)	C9—C10—C23—O2	−166.16 (12)
C3—C4—C5—C6	0.0 (3)	C11—C10—C23—O2	77.62 (13)
N1—C1—C6—C7	1.8 (2)	C9—C10—C23—C24	−37.74 (17)
C2—C1—C6—C7	−179.45 (16)	C11—C10—C23—C24	−153.97 (12)
N1—C1—C6—C5	−177.84 (16)	C9—C10—C23—C22	75.45 (14)
C2—C1—C6—C5	0.9 (3)	C11—C10—C23—C22	−40.78 (13)
C4—C5—C6—C7	179.71 (19)	N2—C22—C23—O2	10.48 (18)
C4—C5—C6—C1	−0.6 (3)	C30—C22—C23—O2	135.09 (13)
C1—C6—C7—C8	−1.6 (2)	C12—C22—C23—O2	−99.88 (14)
C5—C6—C7—C8	178.07 (17)	N2—C22—C23—C24	−113.61 (13)
C6—C7—C8—C9	0.4 (2)	C30—C22—C23—C24	11.00 (15)
C6—C7—C8—C13	−179.82 (15)	C12—C22—C23—C24	136.03 (12)
C7—C8—C9—N1	0.7 (2)	N2—C22—C23—C10	126.36 (12)
C13—C8—C9—N1	−179.07 (14)	C30—C22—C23—C10	−109.03 (13)
C7—C8—C9—C10	−177.45 (14)	C12—C22—C23—C10	16.00 (14)
C13—C8—C9—C10	2.8 (2)	O2—C23—C24—C25	53.0 (2)
N1—C9—C10—C11	−147.13 (13)	C10—C23—C24—C25	−72.5 (2)
C8—C9—C10—C11	31.08 (18)	C22—C23—C24—C25	175.69 (16)
N1—C9—C10—C23	101.00 (15)	O2—C23—C24—C29	−131.95 (14)
C8—C9—C10—C23	−80.79 (17)	C10—C23—C24—C29	102.54 (15)
C9—C10—C11—C12	−68.47 (14)	C22—C23—C24—C29	−9.23 (16)
C23—C10—C11—C12	50.76 (13)	C29—C24—C25—C26	−0.6 (2)
C10—C11—C12—C13	74.78 (14)	C23—C24—C25—C26	173.99 (17)
C10—C11—C12—C14	−155.85 (12)	C24—C25—C26—C27	0.3 (3)
C10—C11—C12—C22	−39.94 (13)	C25—C26—C27—C28	0.1 (3)
C7—C8—C13—O1	3.8 (2)	C26—C27—C28—C29	−0.2 (3)
C9—C8—C13—O1	−176.44 (15)	C26—C27—C28—C33	−179.0 (2)
C7—C8—C13—C12	−176.11 (14)	C33—C28—C29—C24	178.85 (18)
C9—C8—C13—C12	3.6 (2)	C27—C28—C29—C24	−0.2 (3)
C11—C12—C13—O1	137.38 (16)	C33—C28—C29—C30	0.1 (3)
C14—C12—C13—O1	6.6 (2)	C27—C28—C29—C30	−178.96 (17)
C22—C12—C13—O1	−111.69 (17)	C25—C24—C29—C28	0.6 (3)
C11—C12—C13—C8	−42.68 (17)	C23—C24—C29—C28	−175.18 (15)
C14—C12—C13—C8	−173.50 (13)	C25—C24—C29—C30	179.47 (15)
C22—C12—C13—C8	68.25 (15)	C23—C24—C29—C30	3.71 (19)
C13—C12—C14—C15	119.48 (15)	C28—C29—C30—C31	0.5 (3)
C11—C12—C14—C15	−6.12 (19)	C24—C29—C30—C31	−178.42 (17)
C22—C12—C14—C15	−120.61 (14)	C28—C29—C30—C22	−177.26 (16)
C13—C12—C14—C21	−116.48 (15)	C24—C29—C30—C22	3.85 (19)
C11—C12—C14—C21	117.93 (15)	N2—C22—C30—C31	−64.5 (3)
C22—C12—C14—C21	3.44 (16)	C12—C22—C30—C31	57.3 (3)
C21—C14—C15—C16	155.66 (16)	C23—C22—C30—C31	173.5 (2)
C12—C14—C15—C16	−86.58 (19)	N2—C22—C30—C29	112.83 (15)
C21—C14—C15—C20	−25.3 (2)	C12—C22—C30—C29	−125.44 (14)
C12—C14—C15—C20	92.51 (19)	C23—C22—C30—C29	−9.17 (17)
C20—C15—C16—C17	1.8 (3)	C29—C30—C31—C32	−0.3 (3)
C14—C15—C16—C17	−179.07 (18)	C22—C30—C31—C32	176.8 (2)
C20—C15—C16—Cl1	−178.95 (14)	C30—C31—C32—C33	−0.4 (4)
C14—C15—C16—Cl1	0.2 (2)	C31—C32—C33—C28	1.0 (4)
C15—C16—C17—C18	−1.3 (4)	C29—C28—C33—C32	−0.8 (3)
Cl1—C16—C17—C18	179.4 (2)	C27—C28—C33—C32	178.1 (2)
C16—C17—C18—C19	−0.6 (4)	C8—C9—N1—C1	−0.5 (2)
C17—C18—C19—C20	1.8 (4)	C10—C9—N1—C1	177.65 (13)
C18—C19—C20—C15	−1.3 (4)	C2—C1—N1—C9	−179.52 (16)
C16—C15—C20—C19	−0.5 (3)	C6—C1—N1—C9	−0.8 (2)
C14—C15—C20—C19	−179.64 (19)	C14—C21—N2—C34	170.33 (16)
C15—C14—C21—N2	96.03 (16)	C14—C21—N2—C22	42.96 (17)
C12—C14—C21—N2	−27.37 (17)	C30—C22—N2—C21	90.75 (16)
C13—C12—C22—N2	144.56 (12)	C12—C22—N2—C21	−39.21 (15)
C11—C12—C22—N2	−102.35 (13)	C23—C22—N2—C21	−151.50 (14)
C14—C12—C22—N2	21.13 (15)	C30—C22—N2—C34	−34.3 (2)
C13—C12—C22—C30	16.47 (17)	C12—C22—N2—C34	−164.24 (16)
C11—C12—C22—C30	129.56 (13)	C23—C22—N2—C34	83.47 (19)
C14—C12—C22—C30	−106.96 (15)		

### Hydrogen-bond geometry (Å, º)

*Cg*1 is the centroid of the N1/C1/C6–C9 ring.

**Table:**

*D*—H···*A*	*D*—H	H···*A*	*D*···*A*	*D*—H···*A*
O2—H2*A*···N2	0.82	2.12	2.664 (2)	124
C25—H25···N1^i^	0.93	2.59	3.503 (2)	169
C33—H33···O1^ii^	0.93	2.40	3.212 (3)	146
C17—H17···*Cg*1^iii^	0.93	2.73	3.553 (3)	147

Symmetry codes: (i) −*x*+1, −*y*+1, −*z*; (ii) −*x*+2, *y*+1/2, −*z*+1/2; (iii) −*x*+1, −*y*, −*z*.
