# Supplementary figures and images for: Crystal structure of 15-(2-chloro­phen­yl)-6b-hy­droxy-17-methyl-6b,7,16,17-tetra­hydro-7,14a-methanona­phtho[1′,8′:1,2,3]pyrrolo­[3′,2′:8,8a]azuleno[5,6-b]quinolin-14(15H)-one
Source: Acta Crystallogr E Crystallogr Commun. 2015 Dec 31;71(Pt 12):o1091–2. doi: 10.1107/S2056989015024767 (PMC4719995; doi:10.1107/S2056989015024767)

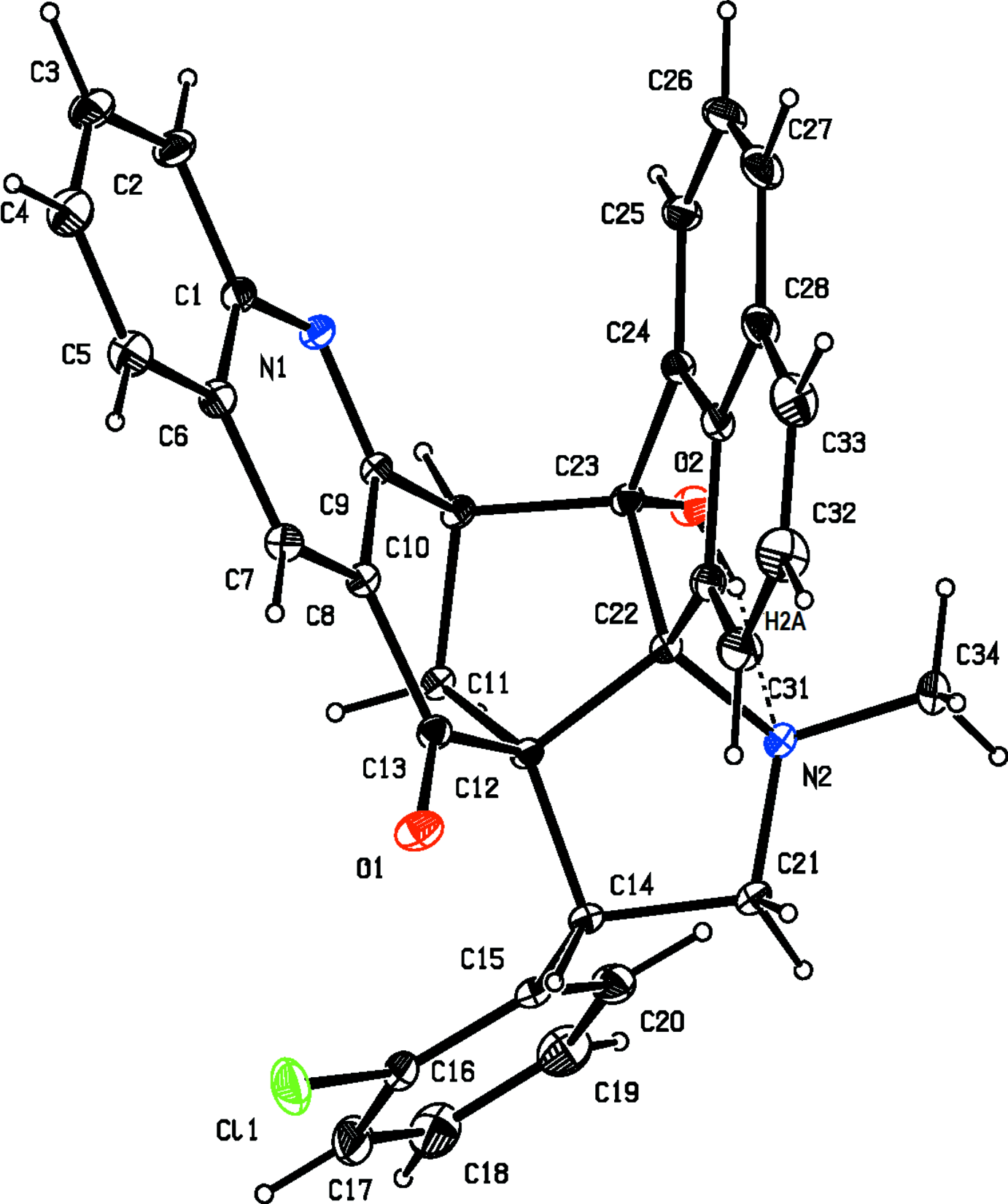

Supplement: Supplementary file 3 [file e-71-o1091-fig1.tif]

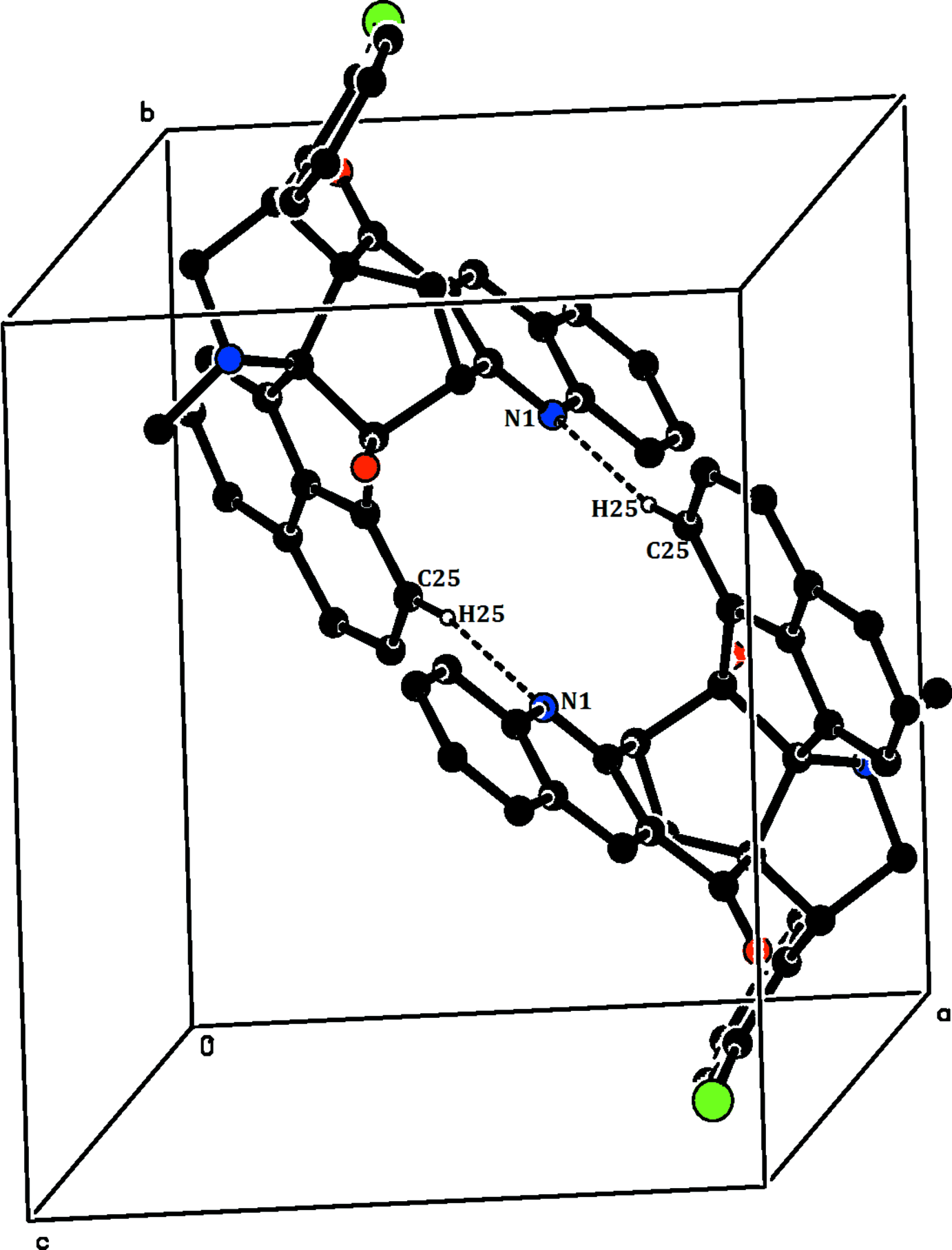

Supplement: Supplementary file 4 [file e-71-o1091-fig2.tif]

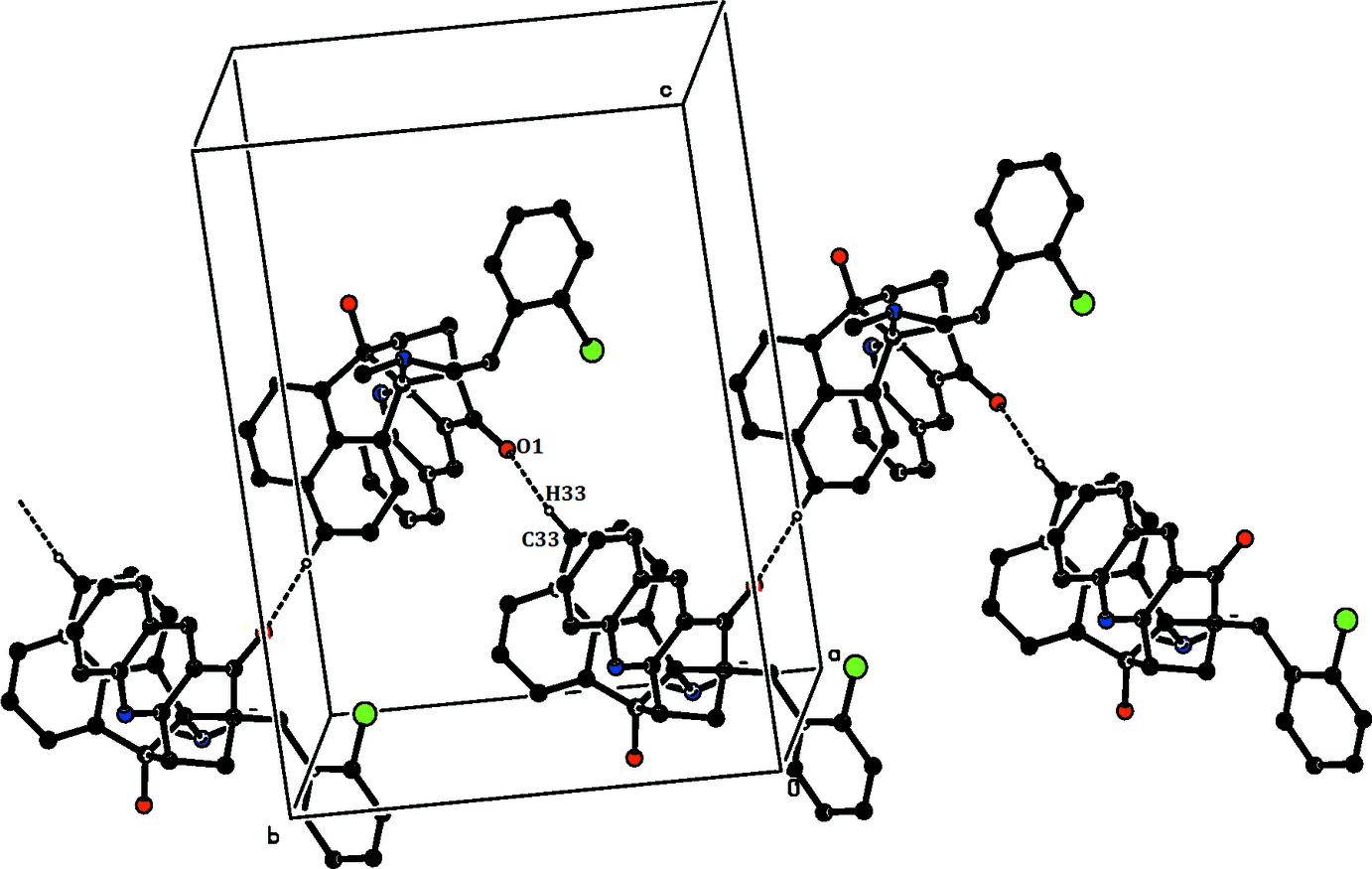

Supplement: Supplementary file 5 [file e-71-o1091-fig3.tif]

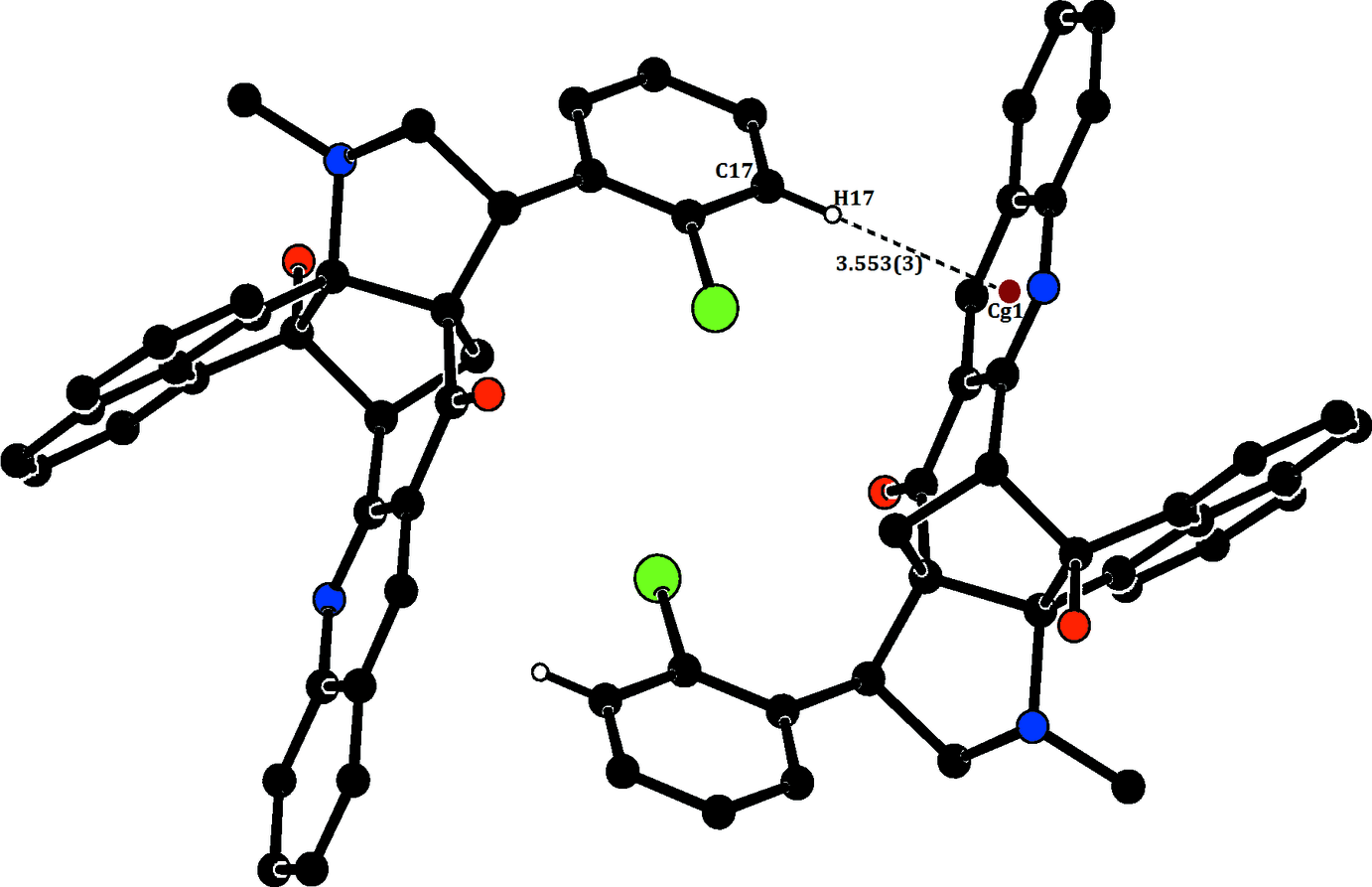

Supplement: Supplementary file 6 [file e-71-o1091-fig4.tif]
